# Cultivation of different seaweed species and seasonal changes cause divergence of the microbial community in coastal seawaters

**DOI:** 10.3389/fmicb.2022.988743

**Published:** 2022-09-07

**Authors:** Ningning Xu, Wenlei Wang, Kai Xu, Yan Xu, Dehua Ji, Changsheng Chen, Chaotian Xie

**Affiliations:** ^1^Fisheries College, Jimei University, Xiamen, China; ^2^Key Laboratory of Healthy Mariculture for the East China Sea, Ministry of Agriculture, Xiamen, China; ^3^Fujian Engineering Research Center of Aquatic Breeding and Healthy Aquaculture, Xiamen, China

**Keywords:** eukaryotic communities, prokaryotic communities, seaweed cultivation, season, interaction, 18S/16S rRNA, microbial community

## Abstract

Although the effects of certain species of seaweed on the microbial community structure have long been a research focus in marine ecology, the response of the microbial community to seasons and different seaweed species is poorly understood. In the present study, a total of 39 seawater samples were collected during 3 months from three zones: *Neoporphyra haitanensis* cultivation zones (P), *Gracilaria lemaneiformis-Saccharina japonica* mixed cultivation zones (G), and control zones (C). These samples were then analyzed using 18S and 16S rRNA gene sequencing to ascertain the fungal and bacterial communities, respectively, along with the determination of environmental factors. Our results showed that increased dissolved oxygen (DO), decreased inorganic nutrients, and released dissolved organic matter (DOM) in seaweed cultivation zone predominantly altered the variability of eukaryotic and prokaryotic microbial communities. Certain microbial groups such as *Aurantivirga*, *Pseudomonas*, and *Woeseia* were stimulated and enriched in response to seaweed cultivation, and the enriched microorganisms varied across seaweed cultivation zones due to differences in the composition of released DOM. In addition, seasonal changes in salinity and temperature were strongly correlated with microbial community composition and structure. Our study provides new insights into the interactions between seaweed and microbial communities.

## Introduction

Marine bacteria, which are ubiquitous in the ocean environment, can form communities with unique functions and structures that play key roles in recycling dissolved nutrients and mediating the global biogeochemical flux of carbon (C), nitrogen (N), phosphorus (P), and sulfur (S) (Falkowski et al., 1998). Given the importance of microbes in marine ecosystems, one of the most vital questions in marine ecology is how microbial communities respond to anthropogenic activities. Aquaculture, one of the predominant human activities in marine systems, easily causes environmental pressure; for example, fish mariculture can result in eutrophication of surrounding seawater. The number and abundance of microbial species in the environment surrounding a fish-farming area is generally high, as is the number of pathogenic bacteria (Buller, 2010). Therefore, more attention has been paid to the response of microbial communities to environmental changes and potential bioremediation of polluted environments in mariculture areas (Apprill, 2017; Luo et al., 2020). Different from aquatic animals, seaweed plays an important role in several critical ecosystem services and functions such as photosynthesis, carbon sequestration, and reducing nutrients load (Duarte et al., 2022). This normally attracts beneficial bacterial species to colonize and inhibit pathogenic bacteria (Egan et al., 2013; Singh and Reddy, 2015). Therefore, the interaction between seaweed and the microbial community is a key issue concerning the ecological benefits of seaweed.

As a primary producer in the marine ecosystem, economically important seaweed species such as *Neoporphyra haitanensis*, *Gracilaria lemaneiformis*, and *Saccharina japonica* can generate high levels of productivity through photosynthesis and effectively remove excessive nutrients such as N and P (Yang et al., 2015; Duarte et al., 2022). Wild seaweed form the most extensive and productive vegetated coastal habitats and contribute to C uptake in the global coastal ocean; seaweed is estimated to support a global net primary productivity of about 1.5 Pg C year^–1^ (Duarte et al., 2017). In addition, a recent study confirmed that large-scale cultivation of *S. japonica* could increase the seawater oxygen concentration and buffer seawater acidification (Xiao et al., 2021). These studies suggest that seaweed could play a vital role in the restoration and protection of the marine environment. Seaweed normally attracts and promotes colonization of beneficial bacteria that multiply on the blade surface; in turn, these epiphytic bacteria regulate seaweed development, reproduction, functioning, and counter the intrusion and colonization of harmful bacteria by secreting secondary metabolites and/or antimicrobials (Goecke et al., 2010; Singh and Reddy, 2015). Therefore, significant research has been aimed at studying the bacterial communities associated with seaweed in order to understand the interactions between bacteria and seaweed.

Xie et al. (2017) showed that *G. lemaneiformis* cultivation shifted the diversity, composition, and structure of water and sediment microbial communities in a mariculture system on the coast of Nan’ao Island. In addition, Wang et al. (2020) showed a notable distinction between the microbial communities of seawater with and without *N. haitanensis* cultivation. Not only did the bacterial biodiversity in the culture zones of *Sargassum fusiforme*, *Neopyropia yezoensis*, *Sargassum incisifolium*, and *Phyllospora comosa* show significant differences, but it was also greatly influenced by abiotic factors (Selvarajan et al., 2019; Ahmed and Khurshid, 2021; Zhang et al., 2021; Wood et al., 2022). These results indicated that seaweed cultivation has a strong capacity to shape the bacterial community of seawater. Previous studies have focused on the effects of one natural or cultivated seaweed species on microbial community structure, but the conditions in nature are much more complex: whether in a natural ecosystem or in aquaculture, several seaweed species can coexist in the same sea area. To date, little is known about the response of eukaryotic and prokaryotic microbial communities to the comprehensive large-scale cultivation of a variety of seaweed species.

The present study used high-throughput sequencing based on 16S and 18S rRNA genes to assess the diversity and structure of prokaryotic and eukaryotic microbial communities in the seawater with cultivated *N. haitanensis*, *G. lemaneiformis*-*S. japonica* mixed, and a control zone during different seasons. This study provides useful information for comprehensive understanding of the impact of seaweed cultivation on microbial communities as well as their feedback to seasonal dynamics. The results will promote future studies on the relationship between seaweed and the microbial community in the coastal waters.

## Materials and methods

### Description of sampling sites

The sampling site was located north of Nan’ri Island (25°21′–25°27′N; 119°50′–119°59′E), one of Fujian Province’s largest mariculture bases in south China (Supplementary Figure 1). The cultivation period of *N. haitanensis* is concentrated from the end of September to the second year in February. *G. lemaneiformis* is cultivated from November to May, and *S. japonica* is cultivated in December and harvested in February of the following year. A total of 39 samples were collected in December 2019 and January and May 2020, meaning that 13 samples were collected each month. Four sampling sites were selected in the *N. haitanensis* cultivation zone (P). Six sampling sites were selected in the *G. lemaneiformis*-*S. japonica* mixed cultivation zone (G). Three sampling sites were selected in a control zone without macroalgae cultivation (C). Seawater samples were taken at a depth of ∼30 cm from the seawater surface. The sampling sites and samples were as follows: (i) P, *N. haitanensis* cultivation zone with four samples (P1–4); (ii) G, *G. lemaneiformis*-*S. japonica* mixed cultivation zone with six samples (G1–G6); and (iii) C, another three samples from the open sea adjacent to G but without seaweed cultivation (C1–3). The samples were marked by sampling site and sampling time (Supplementary Table 1).

### Sample collection and environmental data

Seawater samples with a volume of 2.5 L for each sample site were collected in polyethylene containers. Microbes were collected by filtering 1 L of water with 0.22-μm polycarbonate membranes. The filters were then placed into a liquid nitrogen container prior to being stored at −80°C until DNA extraction. Water temperature and salinity were measured on site. The remaining water sample was immediately transported to the refrigerator and subjected to measurements of the following parameters: total nitrogen (TN), total phosphorus (TP), nitrate–nitrogen (NO_3_^–^-N), phosphate–phosphorus (PO_4_^3–^-P), biogenic silica (BSi), chlorophyll *a* (Chla), dissolved organic matter (DOM) (including DOC, DON, DOP: dissolved organic carbon/nitrogen/phosphorous), and POM (including POC, PON, POP: particulate organic carbon/nitrogen/phosphorous). Water samples for TN, TP, NO_3_^–^-N, PO_4_^3–^-P, DOM, and POM measurements were filtered through pre-combusted Whatman GF/F filters (25 mm, 0.7 μm; Whatman, Kent, United Kingdom) under a low vacuum and were determined according to the protocols of Chen et al. (2020). Water samples for chlorophyll *a* (Chl *a*) analysis were filtered through Whatman GF/F filters (25 mm, 0.7 μm), extracted with 90% aqueous acetone, and measured fluorometrically using a spectrophotometer (UV-1800, Shimadzu, Japan). BSi deposited on the acetate microporous filter was analyzed using wet-alkaline digestion method developed by Ragueneau et al. (2005).

### DNA extraction, PCR amplification, and Illumina novaseq 6000 sequencing

Microbial DNA was extracted using the HiPure Soil DNA Kit (Magen, Guangzhou, China) according to the manufacturer’s protocols. The 16S rDNA V3–V4 region of the ribosomal RNA gene was amplified by PCR using the primers 341F: CCTACGGGNGGCWGCAG and 806R: GGACTACHVGGGTATCTAAT. The 18S rDNA V4 region of the ribosomal RNA gene was amplified by PCR using the primers 528F: GCGGTAATTCCAGCTCCAA. PCR reactions were performed at 94°C for 2 min followed by 30 cycles at 98°C for 10 s, 62°C for 30 s, and 68°C for 30 s with a final extension at 68°C for 5 min. PCR reactions were performed in triplicate 50 μl mixtures containing 5 μl of 10 × KOD Buffer, 5 μl of 2 mM dNTPs, 3 μl of 25 mM MgSO_4_, 1.5 μl of each primer (10 μM), 1 μl of KOD Polymerase, and 100 ng of template DNA. Related PCR reagents were from TOYOBO, Japan.

Amplicons were extracted from 2% agarose gels and purified using the AMPure XP Beads (Beckman Agencourt, United States) according to the manufacturer’s instructions, then quantified using an ABI StepOnePlus Real-Time PCR System (Life Technologies, Foster City, CA, United States). Purified amplicons were pooled in equimolar and paired-end sequences (PE250) on an Illumina HiSeq 6000 system (Illumina, CA, United States) at Gene *Denovo* Biological Technology Co., Ltd. (Guangzhou, China). The raw reads were deposited into the NCBI Sequence Read Archive (SRA) database. Bioinformatic analysis was performed using Omicsmart, a dynamic and interactive online platform for data analysis.^1^

### Data processing based on bioinformatics and statistical analysis

Raw reads were further filtered using FASTP. Paired-end clean reads were merged as raw tags using FLASH (version 1.2.11) with a minimum overlap of 10 bp and mismatch error rates of 20%. Noisy sequences of raw tags were filtered under specific conditions to obtain the high-quality clean tags. The clean tags were clustered into operational taxonomic units (OTUs) of ≥97% similarity using the UPARSE (version 9.2.64) pipeline. All chimeric tags were removed using the UCHIME algorithm to finally obtain effective tags for further analysis. The tag sequence with highest abundance was selected as the representative sequence within each cluster. The representative OTU sequences were classified into organisms by a naive Bayesian model using RDP classifier (version 2.2) based on SILVA database (version 132), with the confidence threshold value of 0.8.

The Chao1, Simpson, and other alpha-diversity indexes were calculated in QIIME (V1.9.1) as mentioned above. An OTU rarefaction curve and rank abundance curves were plotted in QIIME. Statistics of the alpha index comparison between groups were calculated by Welch’s *t*-test and the Wilcoxon rank test in R. Alpha index comparisons among groups were computed by Tukey’s HSD test and the Kruskal–Wallis H test in the R project Vegan package (version 2.5.3). For beta diversity analysis, PCA (principal component analysis) was performed using the R project Vegan package (version 2.5.3). Multivariate statistical techniques including PCoA (principal coordinates analysis) and NMDS (non-metric multidimensional scaling) of (Un) weighted UniFrac, Jaccard, and Bray–Curtis distances were generated with the Vegan R package (version 2.5.3) and plotted with the ggplot2 package (version 2.2.1). Statistical tests [Welch’s *t*-test, the Wilcoxon rank test, Tukey’s HSD test, the Kruskal–Wallis H test, Adonis (also called Permanova), and the Anosim test] were performed using the Vegan package in R (version 2.5.3). A related analysis was carried out using the online platform of Gene *Denovo* Biological Technology Co., Ltd. (Guangzhou, China) (see text footnote 1).

## Results

### Effects of seaweed cultivation on chemical properties of seawater

**FIGURE 1:**
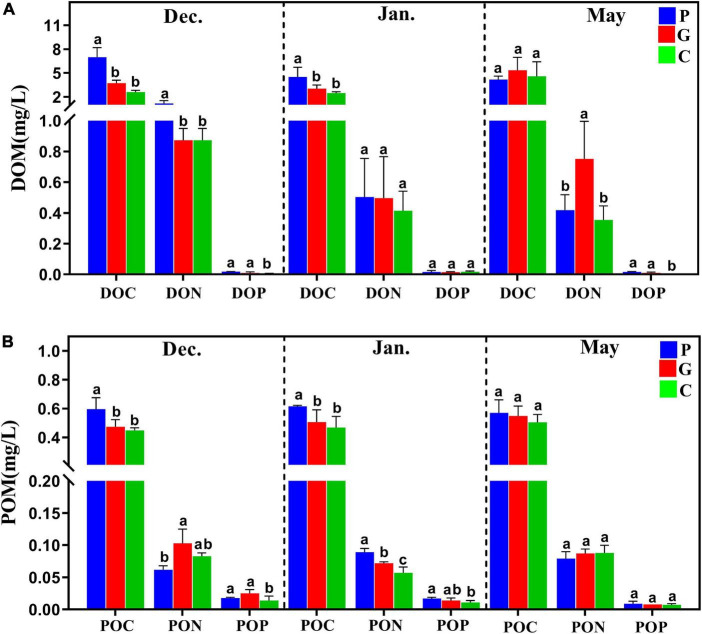
The dissolved organic matter (DOM) and particulate organic matter (POM) released from three study zones during different periods. **(A)** DOC, DON, DOP, dissolved organic carbon/nitrogen/phosphorous. **(B)** POC, PON, POP, particulate organic carbon/nitrogen/phosphorous.

We measured the environmental variables of temperature (T), salinity, NO_3_^–^-N, PO_4_^3–^-P, TN, TP, Chl *a*, BSi, DOM, and POM of water samples (Table 1 and Figure 1). Generally, physicochemical properties of seawater such as temperature and salinity showed clear seasonal patterns. In all three sites, the water temperature gradually decreased from December to January, then increasing in May. In general, the concentrations of NO_3_^–^-N and PO_4_^3–^-P tended to be lower in the seaweed cultivation zones than in the control zone, while TN, TP, Chl *a*, dissolved oxygen (DO), BSi, DOM, and POM showed opposite trends (Table 1). In May, the concentrations of DO, DOM, and POM in the P site were significantly lower than in the G site as a result of complete harvesting of *N. haitanensis*.

**TABLE 1:** Environmental parameters of each sample from December 2019 to May 2020.

	December 2019			January 2020			May 2020		
			
	P	G	C	P	G	C	P	G	C
Temperature (°C)	16.70 ± 0.2^a^	16.73 ± 0.2^a^	16.60 ± 0.3^a^	14.63 ± 0.1^a^	14.53 ± 0.2^a^	14.73 ± 0.12^a^	23.10 ± 0.54^a^	23.13 ± 0.4^a^	23.13 ± 0.46^a^
Salinity (‰)	30.00 ± 0.0^a^	29.83 ± 0.9^a^	29.00 ± 1.0^a^	27.75 ± 0.5^a^	26.83 ± 2.6^a^	28.33 ± 1.53^a^	25.75 ± 1.5^a^	29.0 ± 0.9^b^	26.67 ± 0.89^a^
NO_3_^–^-N (mg/L)	0.48 ± 0.02^b^	0.52 ± 0.05^a^	0.54 ± 0.03^a^	0.53 ± 0.08^a^	0.41 ± 0.05^b^	0.53 ± 0.03^a^	0.33 ± 0.01^b^	0.29 ± 0.04^a^	0.32 ± 0.03^b^
PO_4_^3–^-P (mg/L)	0.028 ± 0.0^a^	0.022 ± 0.0^b^	0.030 ± 0.0^a^	0.033 ± 0.0^a^	0.037 ± 0.0^a^	0.043 ± 0.01^b^	0.028 ± 0.01^a^	0.025 ± 0.0^a^	0.023 ± 0.01^a^
TN (mg/L)	1.72 ± 0.39^a^	1.68 ± 0.16^a^	1.36 ± 0.05^b^	1.13 ± 0.53^ab^	1.58 ± 0.37^a^	1.04 ± 0.26^b^	0.78 ± 0.17^a^	1.60 ± 0.12^b^	0.67 ± 0.08^a^
TP (mg/L)	0.045 ± 0.0^ab^	0.050 ± 0.0^a^	0.040 ± 0.0^b^	0.048 ± 0.0^a^	0.064 ± 0.0^b^	0.06 ± 0.00^ab^	0.042 ± 0.01^a^	0.050 ± 0.0^a^	0.020 ± 0.01^b^
Chl *a* (μg/L)	2.12 ± 0.84^a^	1.51 ± 0.38^a^	1.18 ± 0.20^b^	1.88 ± 0.16^a^	0.97 ± 0.40^b^	0.91 ± 0.18^b^	2.29 ± 0.61^a^	2.09 ± 0.45^a^	1.31 ± 0.93^b^
BSi (μg/L)	8.03 ± 0.82^b^	9.31 ± 0.78^a^	6.56 ± 0.52*^c^*	9.43 ± 2.63^ab^	11.53 ± 2.3^a^	7.93 ± 0.90^b^	12.73 ± 4.22^a^	11.02 ± 1.6^ab^	8.04 ± 2.23^b^
DO (mg/L)	12.71 ± 1.1^a^	14.66 ± 1.5^a^	9.98 ± 1.54^b^	11.85 ± 1.45^a^	13.27 ± 0.98^a^	9.93 ± 0.80^b^	8.88 ± 1.01^b^	12.39 ± 1.10^a^	9.73 ± 0.30^b^

Physical and chemical parameters of surface water at the three sampling zones. P, surface water of N. *haitanensis* cultivation zone; G, surface water of *G. lemaneiformis-S. japonica* mixed cultivation zone; C, surface water of control zone. Different letters (a, b, c) represent significant differences (*p*-value < 0.05) in mean value among different water samples by ANOVA.

### General descriptions of 16 and 18S rRNA gene amplicons

A total of 39 microbial communities present during three sampling periods were analyzed by MiSeq sequencing of 16S rRNA and 18S rRNA gene amplicons. We obtained 4,040,388 high-quality eukaryotic sequences reads and 3,855,691 prokaryotic sequence reads based on the 18S rRNA and 16S rRNA genes, respectively. Operational taxonomic units were defined based on 97% identity, 18S and 16S reads were clustered into 28,020 and 67,039 OTUs, respectively (Supplementary Table 2). Most of the rarefaction curves for the samples reached saturation (Supplementary Figure 2), suggesting sufficient sequencing depth for this study.

### Overall microbial community diversity and structure

**FIGURE 2:**
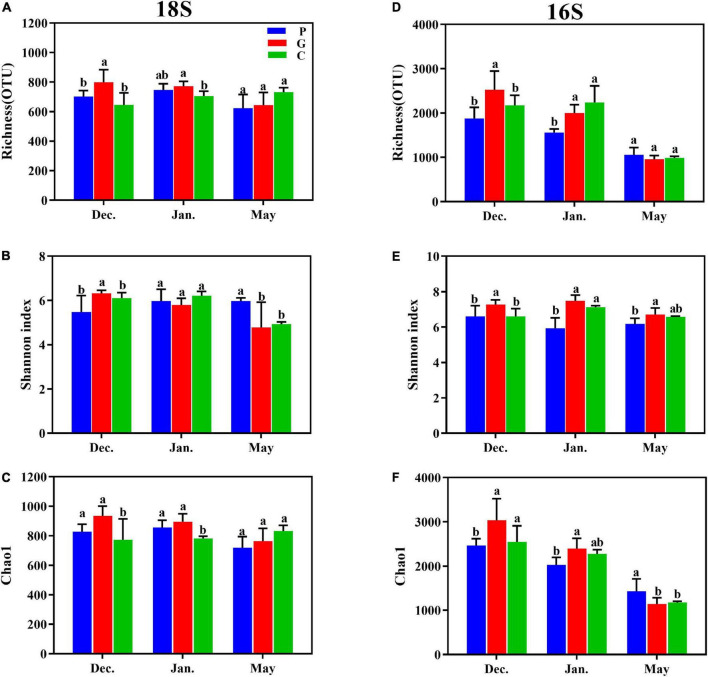
Effects of seaweed cultivation on the alpha-diversity indexes. The observed number of OTUs based on 18S **(A)** and 16S **(D)**, the Shannon index based on 18S **(B)** and 16S **(E)**, and the Chao1 index based on 18S **(C)** and 16S **(F)** shows the alpha diversity of microbial communities across different cultivation periods at the three study sites. Different letters (a, b) represent significant differences (*p*-value < 0.05) in mean value among zones C, P, and G by ANOVA.

After blasting against NCBI using BLASTN, all OTUs were classified into seven groups based on 18S: Viridiplantae (7.81–46.16%), Stramenopiles (7.91–35.47%), Metazoa (9.77–28.17%), Fungi (0.81–12.47%), Alveolata (2.79–11.21%), Rhizaria (1.20–4.47%) and unclassified, while three groups based on 16S were Bacteria (95.46–99.78%), Archaea (0.22–4.54%), and unclassified. The alpha diversity of the eukaryotic and bacterial communities in evenness and richness showed zonal and seasonal differences between cultivation and control samples (Figure 2 and Supplementary Figure 3). As shown in Figure 2, significant differences in the Shannon and Chao1 indexes were observed between zone C and zones P or G whether in eukaryotes and bacteria, especially the biodiversity in zone G that was significantly higher than in zones C and P from December to January, although no differences were observed in May. In May, the abundance of the bacterial community decreased significantly (*p*-value < 0.05) due to the harvesting of *N. haitanensis* and seasonal change (Figures 2D,F).

### Changes of dominant microbial taxa in response to seaweed cultivation

**FIGURE 3:**
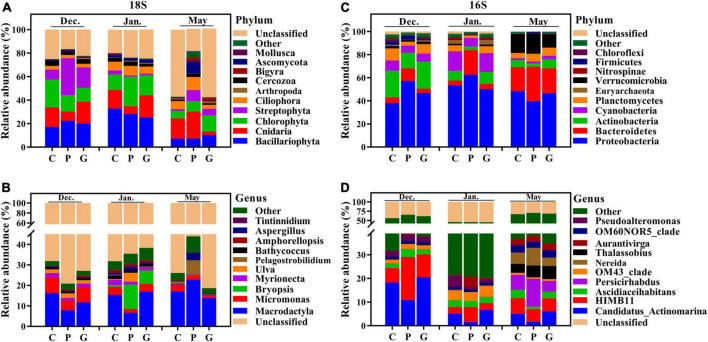
Microbial community composition across different cultivation periods at the three study sites. Relative abundance of different phyla **(A)** and genera **(B)** based on 18S and different phyla **(C)** and genera **(D)** based on 16S in the 39 samples. The abundances are presented in terms of percentages of total effective sequences in a sample at the different levels.

In terms of the relative abundance and taxonomic level, the eukaryotic and bacterial communities in the cultivation zones showed different profiles compared to the control zone. For the eukaryotic community, the enriched phyla were Bacillariophyta, Chlorophyta, and Cnidaria, but their proportions differed among the three study zones (Figure 3A). In December, the relative abundance of Streptophyta (*p*-value < 0.05) was higher than during the other 2 months, and the relative abundance at P was significantly higher (*p*-value < 0.05) than in C and G. In May, the relative abundances of Mollusca, Bigyra, Ascomycota, and Ciliophora were higher (*p*-value < 0.05) at P than at C and G (Figure 3A). For the bacterial community, Proteobacteria was the dominant phylum among all the seawater samples (Figure 3C). During the *N. haitanensis* cultivation period, the abundances of Actinobacteria and Planctomycetes at G and C were significantly higher than at P, while the abundance of Bacteroidetes at P was higher than at G and C (*p*-value < 0.05). Verrucomicrobia was present at greater levels in May than in other periods (Figure 3C).

Although the microbial communities had similarities at the phylum level, the eukaryotic and bacterial communities were strikingly distinct at lower taxonomic levels. For eukaryotic microbes, the most abundant genera were *Macrodactyla* and *Micromonas*. From December to January, higher abundances of *Micromonas* were observed at C and G (*p*-value < 0.05) than at P. The relative abundances of *Bryopsis* and *Pelagostrobilidium* were higher in January and May, respectively, than in other periods, especially at P (Figure 3B). For bacterial microbes, the relative abundances of *Persicirhabdus*, *Thalassobius*, *Nereida*, and *Aurantivirga* were higher in May than in other periods. The relative abundance of *Candidatus-Actinomarina* was high at C and G compared with P, while *HIMB11* was present at a greater level at P than at C and G during the *N. haitanensis* cultivation period (Figure 3D). These results showed a clear dissimilarity of microbial community structure at the three study zones in response to different periods.

### Similarity analyses

**FIGURE 4:**
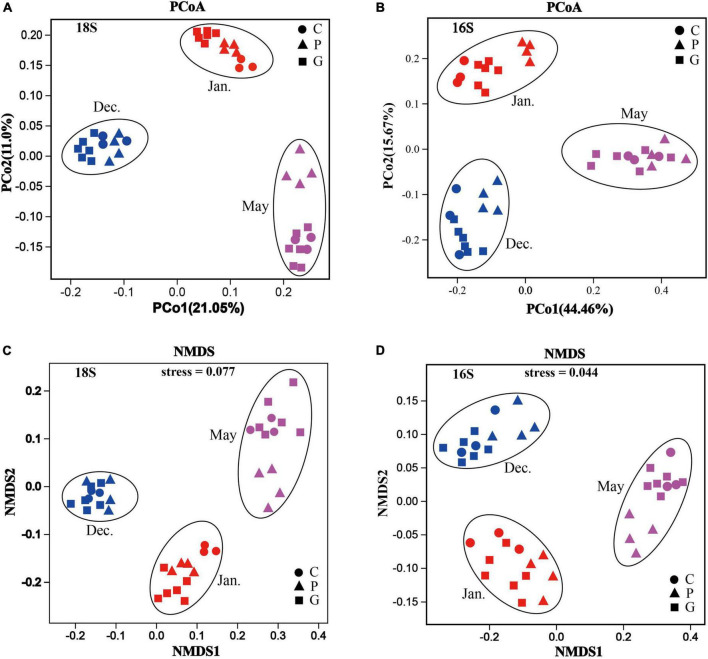
Microbial community structures and identification of patterns based on Bray–Curtis distances in different zones during the various cultivation stages. Principal coordinates analysis (PCoA) plot: all samples (zones C, P, and G) taken in different months based on 18S rRNA genes **(A)** and 16S rRNA genes **(B)** of all the OTUs. Non-metric multi-dimensional scaling analysis (NMDS) plot: all samples (areas C, P, and G) taken in different months based on 18S rRNA genes **(C)** and 16S rRNA genes **(D)** of all the OTUs.

To further determine the differences in microbial community structure in response to seaweed cultivation and seasonal period, a dissimilarity test was performed using Permanova (Adonis) across different zones and periods. For both eukaryotic and bacterial communities, significant differences (*p*-value < 0.05) in community structure were observed among zones C, P, and G in the same period. Similar results were observed in the same study zone among the 3 months (Table 2). For beta diversity interpretation, principal coordinates analysis, PCoA and non-metric multi-dimensional scaling, NMDS was conducted to evaluate similarities among different samples at the OTU level (Figure 4). The results of PCoA showed that all samples formed three clusters. Seawater samples from the same month clustered together, whereas seawater samples from different periods were distantly related with each other in all months (Figures 4A,B), and the results are illustrated in the NMDS plot (Figures 4C,D). Additionally, it should be noted that an NMDS analysis based on each month revealed that the samples of zone P formed a tight cluster that was clearly separated from the other cluster formed by samples from zone C and G (Supplementary Figure 4).

**TABLE 2:** Dissimilarity tests of microbial communities of zones C, P, and G across different periods by ADONIS.

Grouping by sample periods and zones	18S		16S	
		
	*R*	*p*	*R*	*p*
**C**-D vs. **P**-D vs. **G**-D	0.481	0.002	0.551	0.003
**C**-J vs. **P**-J vs. **G**-J	0.767	0.001	0.551	0.001
**C**-M vs. **P**-M vs. **G**-M	0.523	0.006	0.389	0.008
**C**-D vs. **C**-J vs**. C**-M	1	0.006	0.984	0.006
**P**-D vs. **P**-J vs. **P**-M	1	0.001	0.995	0.001
**G**-D vs. **G**-J vs. **G**-M	1	0.001	0.997	0.001

**C**-D, samples of area C in December; **P**-D, samples of area P in December; **G**-D, samples of area G in December; **C**-J, samples of area C in January; **P**-J, samples of area P in January; **G**-J, samples of area G in January; **C**-M, samples of area C in May; **P**-M, samples of area P in May; **G**-M, samples of area G in May. The closer the R-value is to 1, the greater the distance between groups relative to the distance within groups. *p*-Value < 0.05 indicates significant differences between and within groups.

**FIGURE 5:**
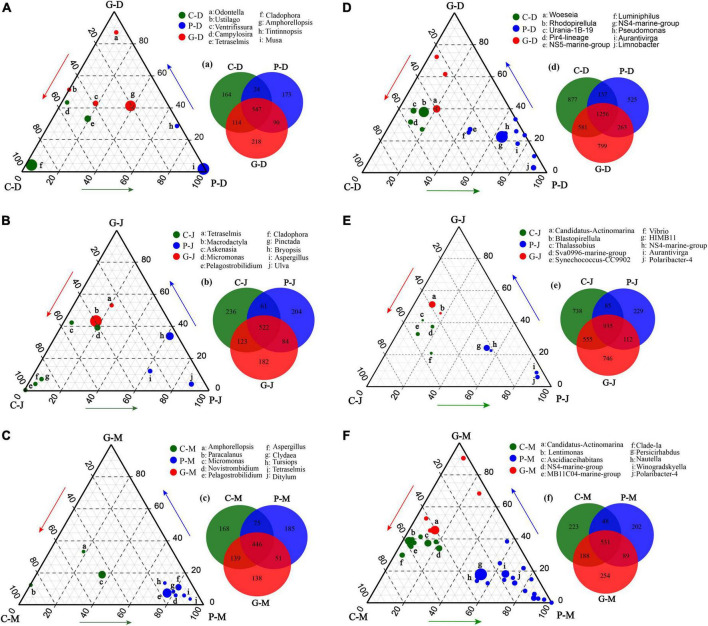
Ternary plot derived from all studied zones and periods based on 18S **(A–C)** and 16S **(D–F)**, and numbers of differentially enriched OTUs among different sampling zones (a–g). Ternary plots of the 10 most abundant genera in seawater. For each plot, the group was taken into consideration, and a density area was drawn to represent similarity within groups. Each circle represents one genus. The size of each circle represents its relative abundance. The position of each circle is determined by the contribution of the indicated compartments to the total relative abundance. Green, blue, and red circles mark genera significantly enriched in zones C, P, and G, respectively (*p*-value < 0.05). The endpoints of each ternary plot represent the sampling zones of their respective periods, and the arrows indicate the direction of the increment.

To identify eukaryotic and bacterial microbes responsible for the diversification among the three sampling zones, we employed relative abundance data based on the unique OTUs to detect the differences in genera among the groups (Figures 5A–F). Using this approach, we identified three distinct eukaryotic and bacterial communities thriving at areas C, P, and G. In addition, we found that there were significant differences in the enriched genera at the same sampling zone in different periods (*p*-value < 0.05).

### Relationships between microbial community structures and seawater properties

**FIGURE 6:**
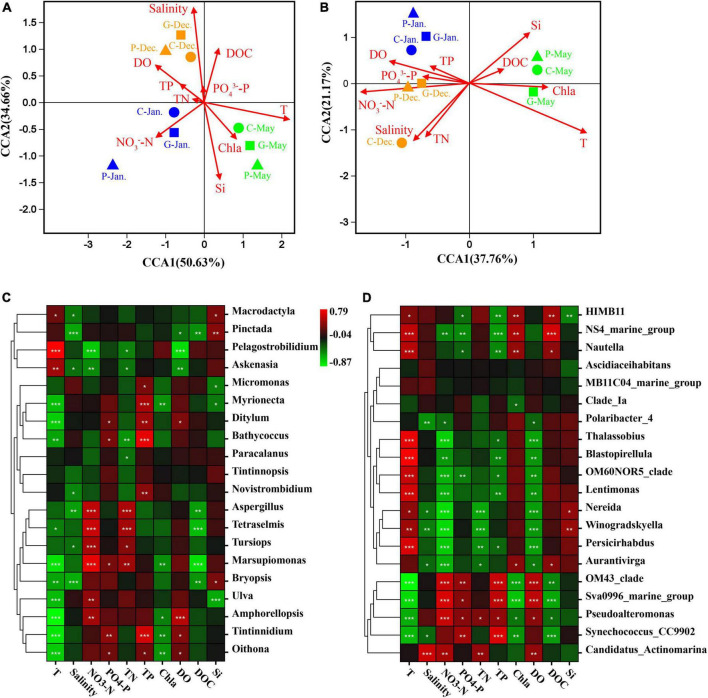
Canonical correspondence analysis (CCA) biplot for the distribution of eukaryotic plankton **(A)** and prokaryotic plankton **(B)** communities with environmental variables. Triangles, samples from area P; squares, samples from area G; circles, samples from area C. Brown, samples in December 2019; blue, samples in January 2020; green, samples in May 2020. T, temperature; TP, total phosphorus; TN, total nitrogen; Si, reactive silicate; DO, dissolved oxygen; DOC, dissolved organic carbon; Chla, chlorophyll *a*. Spearman’s correlation analysis between seawater environmental factors and eukaryotic community **(C)** or prokaryotic community **(D)**. The corresponding intermediate heat-map value is the Spearman correlation coefficient r, *r* > 0: a positive correlation, *r* < 0: negative correlation. The symbols *, **, and *** indicate significance, where *p*-value < 0.05, *p*-value < 0.01, and *p*-value < 0.001, respectively.

Heatmap and correlation coefficient were generated using Omicsmart, a dynamic real-time interactive online platform for data analysis (see text footnote 1). Canonical correspondence analysis (CCA) was implemented to highlight the effects of environmental factors on the microbial community structure at the OTU level (Figures 6A,B). The CCA results showed that the eukaryotic and prokaryotic microbial communities in December, January, and May clustered in groups. The eukaryotic and prokaryotic microbial communities were regulated by multiple environmental variables. Temperature (*R*^2^ = 0.517, *p* = 0.001), silicate (*R*^2^ = 0.270, *p* = 0.003), salinity (*R*^2^ = 0.389, *p* = 0.001), DO (*R*^2^ = 0.232, *p* = 0.003), and NO_3_^–^-N (*R*^2^ = 0.173, *p* = 0.003) were four significant environmental factors affecting the eukaryotic community (Figure 6A). Temperature (*R*^2^ = 0.647, *p* = 0.001), silicate (*R*^2^ = 0.256, *p* = 0.008), salinity (*R*^2^ = 0.299, *p* = 0.002), DO (*R*^2^ = 0.302, *p* = 0.001), TN (*R*^2^ = 0.218, *p* = 0.008), and NO_3_^–^-N (*R*^2^ = 0.7215, *p* = 0.037) were significantly correlated with bacterial communities (Figure 6B). Our results showed that DO was negatively correlated with Chla and reactive silicate, indicating that seaweed cultivation could decrease the density of phytoplankton (Figure 6).

Spearman’s correlation analysis showed that some microbial species could be regulated by different environmental factors. Our results showed stronger negative correlations of most eukaryotic communities with temperature and salinity, while prokaryotic communities were positively correlated with temperature (Figures 6C,D). *Aspergillus*, *Tetraselmis*, *Tursiops*, and *Marsupiomonas* were positively correlated with NO_3_^–^-N and TN, while they exhibited strong negative correlations with DOC (Figure 6C, *p*-value < 0.05). *OM43-clade*, *Sva0996*, and *Pseudoalteromonas* were positively correlated with NO_3_^–^-N, PO_4_^3–^-P, and DO concentrations, whereas they showed strongly negative correlations with DOC and temperature (Figure 6D, *p*-value < 0.05). In addition, many other genera were also strongly correlated with seawater properties, showing both positive and negative correlations.

## Discussion

### Bioremediation potential of seaweed cultivation for environmental improvement

Seaweed cultivation is an environmentally beneficial model of mariculture, and it has introduced co-culturing with animals as an integrated multi-trophic aquaculture (IMTA) system that can reduce nutrient effluents and pollutants as a result of the high bioremediation efficiency (Chai et al., 2018; Xiao et al., 2021). For example, this study found that cultivation of *N. haitanensis*, *G. lemaneiformis*, and S. *japonica* improved water quality by removing NO_3_^–^-N and PO_4_^3–^-P and increasing the DO concentration (Table 1). This is consistent with recent studies that also reported that seaweed mariculture could mitigate ocean acidification (Krause-Jensen et al., 2016; Xiao et al., 2021). In addition, as one of the most important primary producers in coastal ecosystems, a large portion of the photosynthetic products of seaweed was released into ambient seawater as DOM and POM (Wada and Hama, 2013; Chen et al., 2020). As our results demonstrated, the DOM (including DOC, DON, and DOP) and POM (including POC, PON, and POP) contents tended to be higher in the seaweed cultivation zones than in the control zone without seaweed cultivation (Figure 1). On the one hand, DOC is a source of metabolic energy for microorganism growth and as such enriches the biodiversity of marine ecosystems (Thornton, 2014). On the other hand, partial POC can be suspended in the water column, buried in sediments, or exported to the deep sea, thereby acting as a CO_2_ sink (Duarte et al., 2017; Chen and Xu, 2020).

### Seaweed cultivation altered the microbial community composition and structure

The interactions between seaweed and microorganisms depend on the variation of abiotic factors, as free-living microbial communities are strongly driven by environmental factors such as light, temperature, dispersal limitation, and the chemical environment (Hellweger et al., 2014; Gusareva et al., 2019; Ahmed et al., 2021). Metagenomic sequencing, COG, and SEED annotations showed that microbial community assembly was largely dependent on functions rather than phylogenetic similarity and could adapt to new environments by changing the community structure and composition to form a phycosphere with unique structure and function (Logue and Lindstrom, 2010; Langenheder and Székely, 2011; Semblante et al., 2017; Wang et al., 2020). Our results also identified differences in microbial community structure between seaweed cultivation and control zones, differences that may have been due to the impacts of the seaweed cultivation on the environmental factors (Table 1 and Figure 1). The thalli of *N. haitanensis*, *G. lemaneiformis*, and *S. japonica* can take up CO_2_ and release O_2_ into the seawater so that DO was greatly increased at zones P and G (Table 1). Meanwhile, the CCA results indicated that many microorganisms’ abundances were significantly positively correlated with DO content (Figure 6). Many microbes in the ocean require oxygen for basic metabolism in necessary redox processes, while low concentrations of oxygen could restrict primary production and reduce microbial abundance (Semblante et al., 2017). Thus, higher concentrations of DO resulting from seaweed cultivation in coastal ecosystems could be related to increased phylogenetic and functional diversity of the microbial communities (Bryant et al., 2012).

In addition, the pattern and concentration of nutrients have direct influence on microbial metabolism. For example, nutrients usually increase microbial abundance by facilitating cell growth and division, while decreased concentrations of inorganic nitrogen and phosphorus due to nutrient uptake by seaweed will impact the diversity and composition of microbial communities (Donachie et al., 2001; Xie et al., 2017). Furthermore, organic matter released by algae can be stored, respired as DOC, or put into growth together with other nutrients such as phosphate and nitrogen, then utilized directly by microbes (Anderson and Ducklow, 2001; Jiao et al., 2010). DOC released by seaweed is composed of free amino acids, sugars, and organic acids (Thornton, 2014; Wegley Kelly et al., 2022). Among these organic substances, the algal polysaccharides are potential sources of carbon and energy for various marine bacteria (Goecke et al., 2010; Hehemann et al., 2012). For example, the marine macroalga *Ulva mutabilis* can provide carbon for *Roseovarius* in the form of glycerol (Kessler et al., 2018). Proteobacteria are known to digest galactan sulfates in red algal cell walls (Miranda et al., 2013). Cole et al. (1988) also found that the release of DOC by macroalgae may largely affect the microbial loop; the more DOC exuded by algae, the more bacteria grew in lakes and coastal waters. Herein, we found that DOC released by seaweed had a significant effect on the distributions of eukaryotic and prokaryotic microbial communities (Figure 6).

Additionally, some potential pathogens such as *Vibrio* were abundant at zone C (Figure 5E), implying a healthier community composition at the seaweed cultivation zone (Austin and Zhang, 2010; Hubbard et al., 2016). Such divergence in abundances of genera may be due to the fact that seaweed can defend against microbial and pathogenic invasion by producing a wide variety of secondary metabolites (Goecke et al., 2010). In summary, the decreased concentration of inorganic nutrients, increased DO content, and the release of DOM by seaweed cultivation could be the driving forces shaping the eukaryotic and prokaryotic communities.

### Enriched microbial groups in response to different seaweed cultivation

Seaweeds are known to harbor a variety of bacterial symbionts on and around the plants. Our results were generally consistent with previous studies of marine microbial communities showing that Cyanobacteria, Bacteroidetes, and Proteobacteria were the dominant groups in seawater during different seasons (Gilbert et al., 2010; Fuhrman et al., 2015). In addition, even though the three study zones were geographically adjacent and experienced the same ocean currents and climate conditions during the same periods, the microbial communities in zone C, P, and G showed spatial variation. The ternary plot analyses showed that during the *N. haitanensis* cultivation periods, zone P had larger percentages of *Aurantivirga*, *NS5-marine-group*, *NS4-marine-group*, *Pseudomonas*, *HIMB11*, *Limnobacter*, and *Polaribacter*-4 (Figures 5D,E). *Aurantivirga*, *NS5-marine-group*, *NS4-marine-group*, and *Polaribacter*-4 are members of the family Flavobacteriaceae, bacteria that are considered as potential microorganisms for degrading seaweed fucoidan, a mixture of sulfated fucose-containing polysaccharides. These microbes have the capacity to utilize D-lactose, sucrose, and inositol and to reduce nitrates to nitrites (Sakai et al., 2002; Nedashkovskaya et al., 2013). *Pseudomonas*, *Limnobacter*, and *HIMB11* play important roles in cycling of S, N, and organic compounds in marine ecosystems, and thus have environmentally beneficial potential (Spiers et al., 2000; Lu et al., 2011). Moreover, *Woeseia*, *Candidatus-Actinomarina*, and *Blastopirellula* showed higher abundances at zone G (Figures 5D–F). *Woeseia*, as core members of microbial communities in marine ecosystem, are able to assimilate inorganic carbon (Dyksma et al., 2016). Bacterial communities of *Candidatus-Actinomarina* have already been reported to play a key role in organic matter processing in oceans and have the potential for complex-polymer degradation (López-Pérez et al., 2020; Kopprio et al., 2021). *Blastopirellula* abundance was related to the microbial biomass, nitrogen, and nitrogen mineralization rate (Lee et al., 2013). The above results indicate that the microbial composition in seaweed cultivation zones has been regulated by host-specific processes (Roth-Schulze et al., 2016; Pei et al., 2021; Wood et al., 2022).

Increasing evidence suggests that microbial communities are shaped by strong selective forces arising from their hosts (Yakimov et al., 2006; Reis et al., 2009). On the one hand, as an adhesion substrate, seaweed provides a good living environment for the adhesion of microorganisms in seawater. The three types of seaweed that were utilized in the present study differed significantly in morphology and growth characteristics. A recent study reported that the *S. incisifolium* thalli can attract and accommodate more microorganisms than *Arthrocardia flabellata* can (Selvarajan et al., 2019). In the present study, during the cultivation period in December and January the cultivation area of *G. lemaneiformis-S. japonica* was much larger than that of *N. haitanensis*, which may be one of the main reasons for the higher Chao1 index in the G zone than in the P zone (Figure 2F). DOM is one of the most complex and abundant chemical mixtures, and its composition and concentration are posited to regulate microbial energetics. Previous studies have reported that the released DOM (e.g., proteins, carbohydrates, and lipids) differ markedly among different macroalgae species (Lancelot, 1984; Chen et al., 2020; Lønborg et al., 2020). As our results showed, the DOC release amount in zone P was significantly higher than in zones G and C in December and January (Figure 1); this may have led to various microorganisms in zone P being positively correlated with DOC, such as in *Aurantivirga*, *NS4-marine-group*, *HIMB11*, and *Nautella* (Figures 5D,E, 6D). In addition, the patterns in multivariate exo-metabolites featured variation among different seaweeds. The molecular structures of these compounds are one factor affecting the community of microorganisms that metabolize the compounds (Wegley Kelly et al., 2022). Thus, we suggest that the vast metabolic diversity of natural compounds produced by seaweed may provide a basis for selectively promoting, inhibiting, and recruiting specific microbes to enable the shaping of microbial communities tailored to the seawater properties.

### Seasonal variation of the microbial community

It is worth noting that due to the extension of the culture period, certain environmental factors fluctuated, leading to significant differences in microbial community structure in different seasons (Figure 4). As indicated by the Chao1 index, in the present study the highest eukaryotic and prokaryotic diversity occurred in December, while the lowest appeared in May (Figure 3). PCoA and NMDS analyses also confirmed the diversity of microbiota distributed independently in different periods (Supplementary Figure 3). This observation was similar to previous studies that have highlighted remarkable seasonal diversity and dynamics in marine microbes (Gilbert et al., 2010; Egge et al., 2015; Fu et al., 2020).

The divergence of microbe communities could be interpreted by the seasonally related features in salinity and temperature in the studied areas. The present study found significant variation in temperature and salinity among different periods (Table 1). Eukaryotic and prokaryotic communities had significant positive correlations with salinity in December and positive correlations with temperature in May (Figures 6A,B). It is well known that temperature is a major environmental driver determining microbial populations and their functional activities by effecting metabolic niches (Ian and Smale, 2017; Gestel et al., 2020). For example, warming will decrease total plankton biomass and alter prokaryotic community composition in seawater (Flombaum et al., 2013; Morán et al., 2017). Additionally, a large-scale meta-analysis suggested that salinity was the major determinant across ocean ecosystems, exceeding the influence of temperature (Lozupone and Knight, 2007). This is consistent with a recent study that demonstrated that microalgal community diversity, richness, and evenness decreased with enhancing salinity in lake waters (Yue et al., 2019). In the present study, the salinity level varied seasonally, with the highest levels in December and lowest in May (Table 1). This variation may drive the divergence of microbial abundance and community structure. Accordingly, the crosstalk of environmental factors (primarily including temperature and salinity) caused by seasonal changes and different species of seaweed critically affects the microbial community structure.

## Conclusion

In our research, *N. haitanensis* and *G. lemaneiformis-S. japonica* cultivation increased DO, decreased nutrients, and produced specific compounds (e.g., DOM and POM). These changes may not only lead to improved water quality but also alter the eukaryotic and prokaryotic microbial communities in terms of alpha diversity, composition, and structure. Additionally, different microbial groups were enriched in response to different seaweed cultivation regimes and seasons. Therefore, this study has enhanced our knowledge of the important role of seaweed cultivation in shaping the microbial diversity in seawater.

## Data availability statement

The datasets presented in this study can be found in online repositories. The names of the repository/repositories and accession number(s) can be found below: NCBI, PRJNA850096 and PRJNA851214.

## Author contributions

NX: data curation, experiment, and writing—original draft preparation. WW and KX: methodology and writing—review and editing. CX: conceptualization, methodology, and formal analysis. YX, DJ, and CC: supervision and writing—review and editing. All authors contributed to the article and approved the submitted version.

## Footnotes

1
https://www.omicsmart.com
