# Supplementary material for: Cultivation of different seaweed species and seasonal changes cause divergence of the microbial community in coastal seawaters
Source: Front Microbiol. 2022 Sep 7;13:988743. doi: 10.3389/fmicb.2022.988743 (PMC9490310; doi:10.3389/fmicb.2022.988743)
Supplement: Supplementary file 1 [file Data_Sheet_1.docx]

Supplementary Material

# Table S1 Information of collected samples

| Sample name* | Name of sampling location | Sampling time |
| --- | --- | --- |
| C-D | Offshore (Control) | December 2019 |
| C-J | Offshore (Control) | January 2020 |
| C-M | Offshore (Control) | May 2020 |
| P-D | *Neoporphyra* cultivation | December 2019 |
| P-J | *Porphyra* cultivation | January 2020 |
| P-M | *Porphyra* cultivation | May 2020 |
| G-D | *Gracilaria*-*Saccharina* cultivation | December 2019 |
| G-J | *Gracilaria*-*Saccharina* cultivation | January 2020 |
| G-M | *Gracilaria*-*Saccharina* cultivation | May 2020 |

* C, P and G refers to the abbreviation of sampling location (Control, *Neoporphyra* and *Gracilaria*-*Saccharina*, respectively)；D, J and M refers to the abbreviation of sampling time (December, January and May, respectively).

# Table S2 Detailed description of 16S rRNA gene data set and 18S rRNA gene data set.

| **Sample**  **(18S)** | **OTU number** | | **Total Tags** | |  | **Unclassified Tags** | | | **Singleton Tags** | |
| --- | --- | --- | --- | --- | --- | --- | --- | --- | --- | --- |
|  | **All** | **Mean** | **All** | **Mean** |  | **All** | **Mean** | **All** | | **Mean** |
| **C-D** | 2034 | 678 | 323613 | 107871 |  | 61725 | 20575 | 31176 | | 10392 |
| **C-J** | 2317 | 772 | 299757 | 99919 |  | 42007 | 14002 | 25761 | | 8587 |
| **C-M** | 2196 | 732 | 304634 | 101545 |  | 151481 | 50493 | 21473 | | 7158 |
| **P-D** | 2806 | 702 | 423695 | 105924 |  | 52650 | 13162 | 37715 | | 9429 |
| **P-J** | 2986 | 747 | 407887 | 101972 |  | 67155 | 16789 | 34994 | | 8749 |
| **P-M** | 2497 | 624 | 404348 | 101087 |  | 64207 | 16052 | 13722 | | 3431 |
| **G-D** | 4716 | 786 | 632552 | 105425 |  | 107159 | 17860 | 66294 | | 11049 |
| **G-J** | 4704 | 784 | 614466 | 102411 |  | 116992 | 19499 | 49019 | | 8170 |
| **G-M** | 3764 | 627 | 629436 | 104906 |  | 332870 | 55478 | 35464 | | 5911 |
| **Total** | 28020 | 6452 | 4040388 | 931060 |  | 996246 | 223910 | 315618 | | 72876 |
| **Sample**  **(16S)** | **OTU number** | | **Total Tags** | |  | **Unclassified Tags** | | | **Singleton Tags** | |
|  | **All** | **Mean** | **All** | **Mean** |  | **All** | **Mean** | **All** | | **Mean** |
| **C-D** | 6516 | 2172 | 318458 | 106153 |  | 0 | 0 | 39009 | | 13003 |
| **C-J** | 6715 | 2238 | 283094 | 94365 |  | 0 | 0 | 60684 | | 20228 |
| **C-M** | 2956 | 985 | 299629 | 99876 |  | 2 | 0.7 | 63061 | | 21020 |
| **P-D** | 7515 | 1879 | 396832 | 99208 |  | 0 | 0 | 63329 | | 15832 |
| **P-J** | 6238 | 1560 | 382023 | 95506 |  | 5 | 1.3 | 86146 | | 21537 |
| **P-M** | 4233 | 1058 | 370926 | 92732 |  | 0 | 0 | 97961 | | 24490 |
| **G-D** | 15140 | 2523 | 623726 | 103954 |  | 0 | 0 | 129245 | | 21541 |
| **G-J** | 11995 | 1999 | 587501 | 97917 |  | 5 | 0.8 | 118397 | | 19733 |
| **G-M** | 5731 | 955 | 593502 | 98917 |  | 0 | 0 | 99714 | | 16619 |
| **Total** | 67039 | 15369 | 3855691 | 888628 |  | 9 | 3 | 757546 | | 174003 |


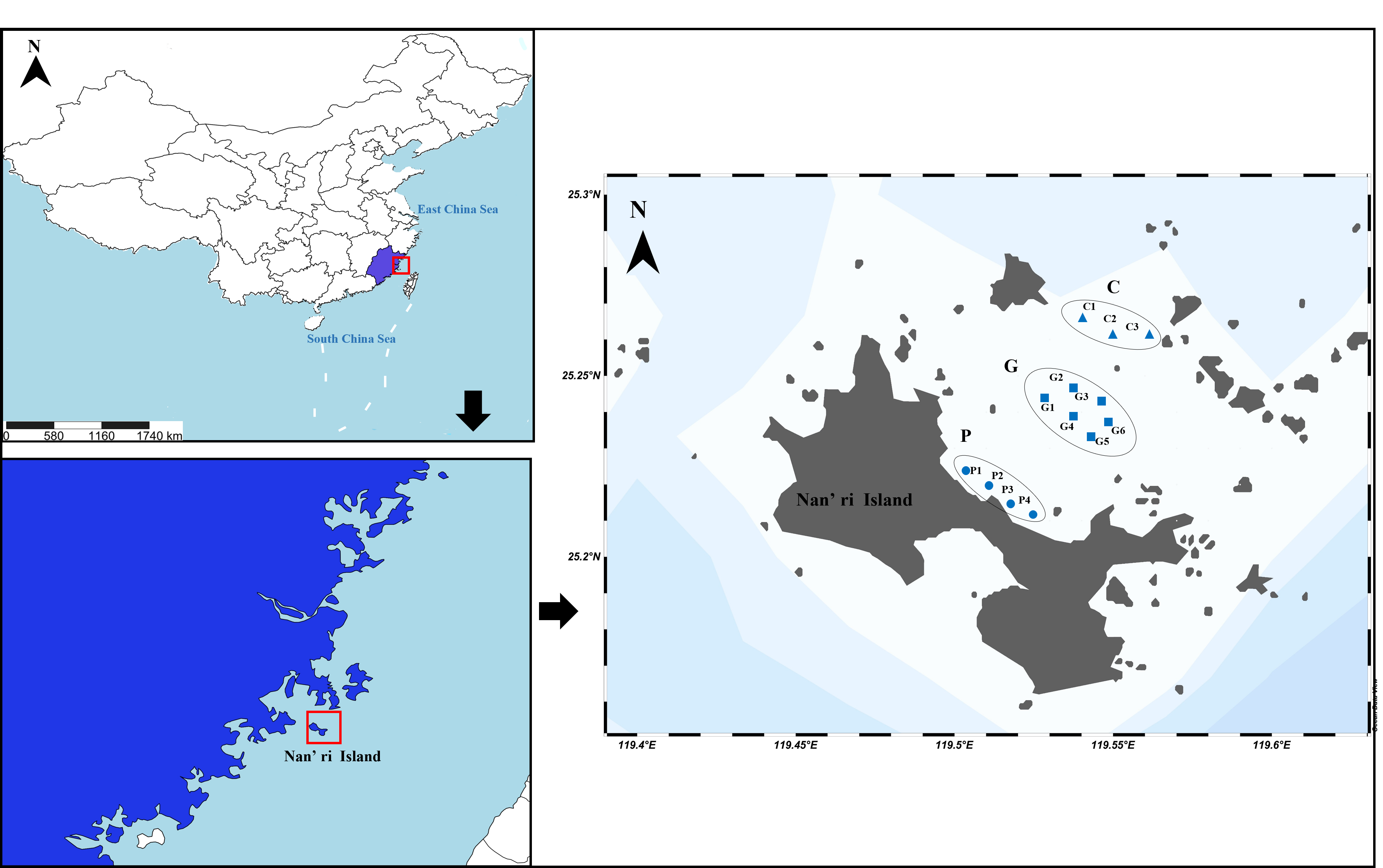


**Figure S1** Location of sampling sites and samples in Nan’ri Island, China. P: *Neoporphyra haitanensis* cultivation zones with four samples (P1-4); G: *Gracilaria lemaneiformis-Saccharina japonica* mixed cultivation zones with six samples (G1-6); C: control zones (without seaweed cultivation) with three samples (C1-3).


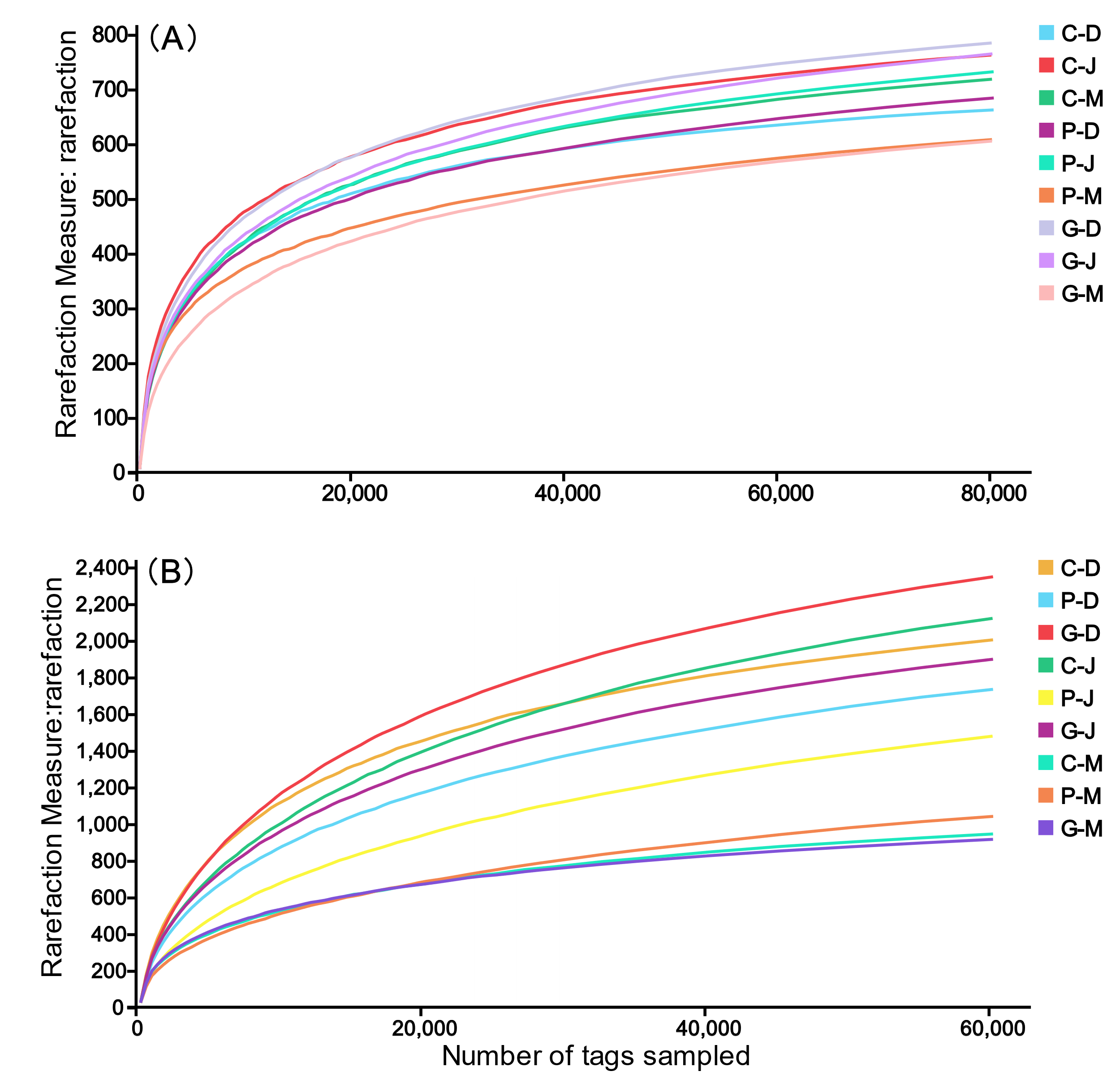


**Figure S2** Rarefaction curves of all samples (A:18S rRNA gene, B:16S rRNA gene)


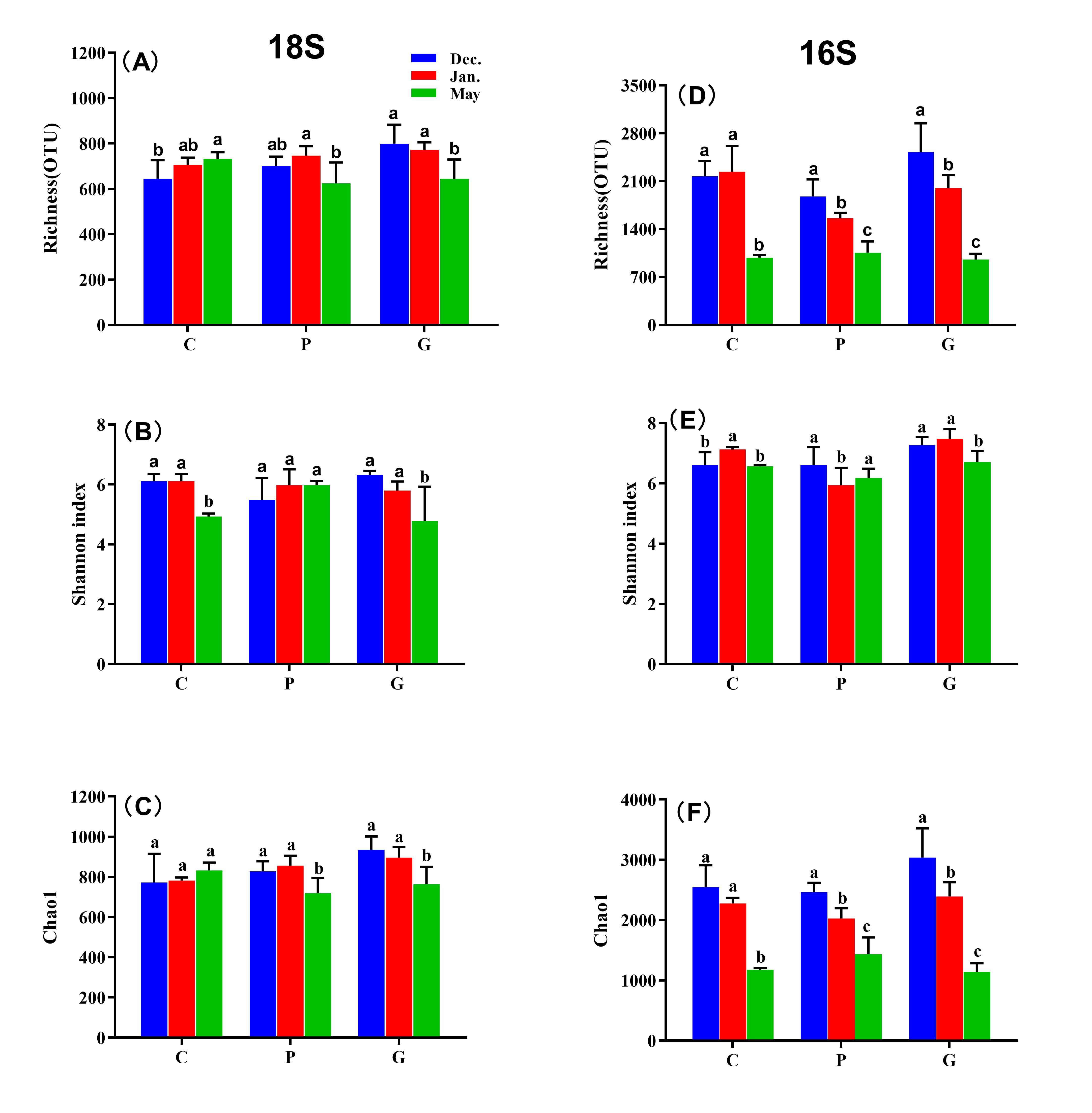


**Figure S3** Effects of seaweed cultivation on the alpha-diversity indices. The observed number of OTUs based on 18S (A) and 16S (D), shannon index based on 18S (B) and 16S (E), and Chao1 based on 18S (C) and 16S (F) showed α-diversity of microbial communities across three study sites (C, P and G) during different cultivation periods. Different letters (a, b, c) represent significant differences (*P* < 0.05) in mean value among zone C, P and G by ANOVA.


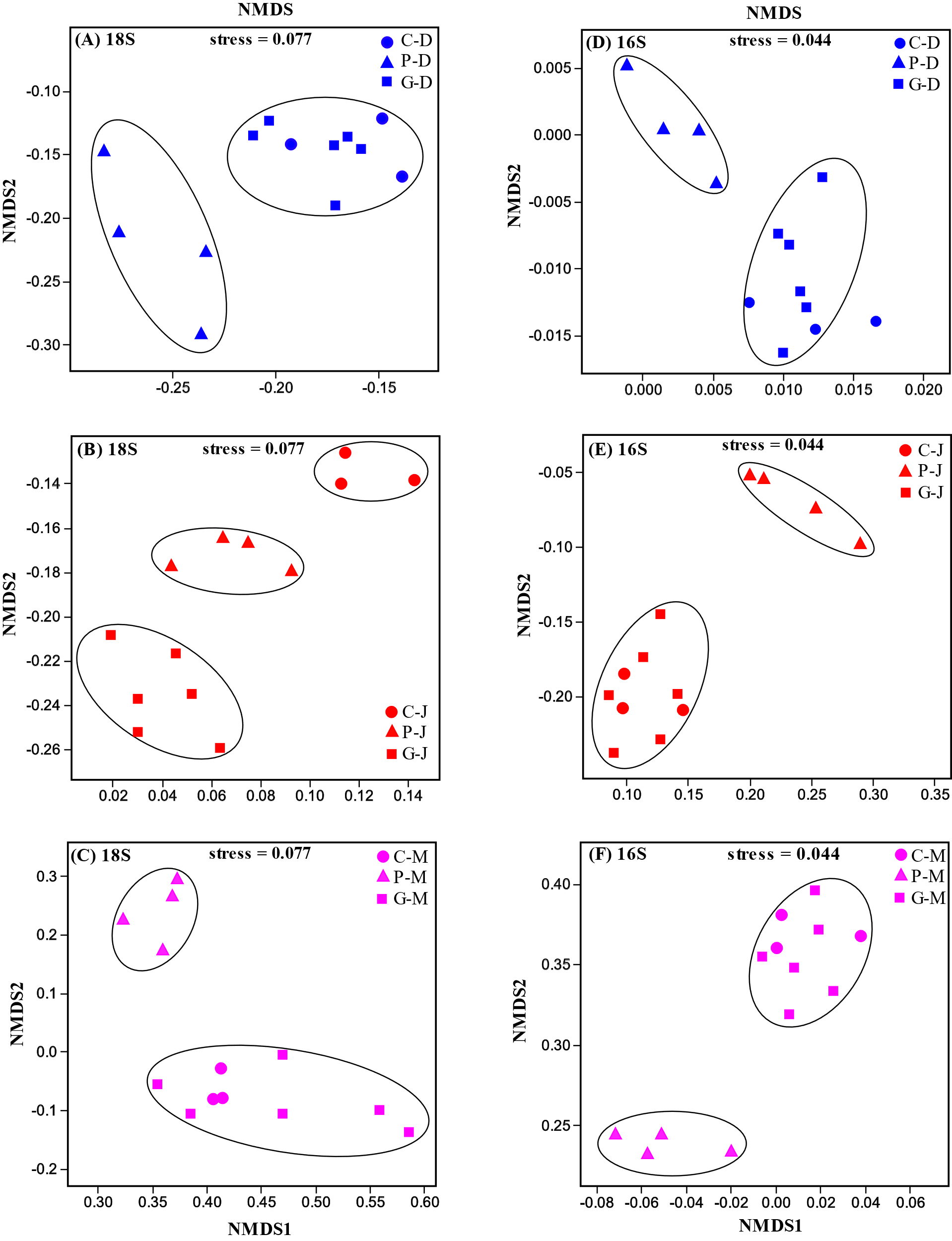


**Figure S4** Nonmetric multi-dimensional scaling (NMDS) plot of seawater samples.
